# Stochastic modeling suggests that noise reduces differentiation efficiency by inducing a heterogeneous drug response in glioma differentiation therapy

**DOI:** 10.1186/s12918-016-0316-x

**Published:** 2016-08-11

**Authors:** Xiaoqiang Sun, Jiajun Zhang, Qi Zhao, Xing Chen, Wenbo Zhu, Guangmei Yan, Tianshou Zhou

**Affiliations:** 1 Zhong-shan School of Medicine, Sun Yat-Sen University, Guangzhou, 510089 China; 2 School of Mathematical and Computational Science, Sun Yat-Sen University, Guangzhou, 510275 China; 3 School of Mathematics, Liaoning University, Shenyang, 110036 China; 4 Research Center for Computer Simulating and Information Processing of Bio-macromolecules of Liaoning Province, Shenyang, 110036 China; 5 School of Information and Electrical Engineering, China University of Mining and Technology, Xuzhou, Jiangsu 221116 China

**Keywords:** Stochastic modeling, Ultrasensitivity, Noise, Differentiation efficiency, Drug resistance, Glioma differentiation therapy

## Abstract

**Background:**

Glioma differentiation therapy is a novel strategy that has been used to induce glioma cells to differentiate into glia-like cells. Although some advances in experimental methods for exploring the molecular mechanisms involved in differentiation therapy have been made, a model-based comprehensive analysis is still needed to understand these differentiation mechanisms and improve the effects of anti-cancer therapeutics. This type of analysis becomes necessary in stochastic cases for two main reasons: stochastic noise inherently exists in signal transduction and phenotypic regulation during targeted therapy and chemotherapy, and the relationship between this noise and drug efficacy in differentiation therapy is largely unknown.

**Results:**

In this study, we developed both an additive noise model and a Chemical-Langenvin-Equation model for the signaling pathways involved in glioma differentiation therapy to investigate the functional role of noise in the drug response. Our model analysis revealed an ultrasensitive mechanism of cyclin D1 degradation that controls the glioma differentiation induced by the cAMP inducer cholera toxin (CT). The role of cyclin D1 degradation in human glioblastoma cell differentiation was then experimentally verified. Our stochastic simulation demonstrated that noise not only renders some glioma cells insensitive to cyclin D1 degradation during drug treatment but also induce heterogeneous differentiation responses among individual glioma cells by modulating the ultrasensitive response of cyclin D1. As such, the noise can reduce the differentiation efficiency in drug-treated glioma cells, which was verified by the decreased evolution of differentiation potential, which quantified the impact of noise on the dynamics of the drug-treated glioma cell population.

**Conclusion:**

Our results demonstrated that targeting the noise-induced dynamics of cyclin D1 during glioma differentiation therapy can increase anti-glioma effects, implying that noise is a considerable factor in assessing and optimizing anti-cancer drug interventions.

**Electronic supplementary material:**

The online version of this article (doi:10.1186/s12918-016-0316-x) contains supplementary material, which is available to authorized users.

## Background

Glioma differentiation therapy is a novel strategy for inducing glioma cells to differentiate into normal-like cells using specific drugs [1]. Although some advances in exploring the molecular mechanisms involved in drug-induced glioma differentiation have been made, a model-based comprehensive analysis is still needed to understand these differentiation mechanisms and improve the effects of anti-cancer therapeutics.

Experimental studies have revealed a variety of signaling pathways that are involved in the regulation of glioma differentiation. It has been shown that the elevation of cAMP levels by cholera toxin (CT) can induce glioma cell differentiation, which is mediated by CREB phosphorylation at Ser-133 in a PKA dependent manner [2]. cAMP/PKA signaling can also inhibit the PI3K/AKT pathway, leading to the activation of the downstream molecule GSK-3β and subsequent degradation of cyclin D1 [3]. Additionally, the IL-6/JAK2/STAT3 pathway, which is activated by increased cAMP levels, is also involved in glioma cell differentiation [4]. In such studies, Glial fibrillary acidic protein (GFAP) is applied as a reliable marker for evaluating the differentiation of glioma cells.

Mathematical models have shown great potential in contributing to the understanding of biological mechanisms and the generation of testable hypotheses or predictions. In a recent study [5], we constructed an ordinary differential equation (ODE) model for the signaling network involved in glioma differentiation which revealed a bi-stable mechanism for phenotype switching during glioma differentiation. On the other hand, extensive stochastic noise exists in signal transduction and phenotypic regulation [6] and biological regulatory systems are dynamic and stochastic. Several studies have demonstrated an intricate interplay between noise and the structure and spatiotemporal dynamics [7] of the signaling network [8, 9] during cancer therapy. However, few studies have examined the relationship between inherent noise and drug efficacy in the induction of glioma differentiation.

In the present study, we adopted glioma differentiation therapy as a realistic case for investigating how the noise that inevitably exists in signaling networks influences drug efficacy and contributes to drug resistance, focusing on the functional role of this noise in the drug response of glioma cancer cells. We developed both an additive noise model (ANM) and a Chemical-Langenvin-Equation (CLE) model to simulate the stochastic dynamics of the signaling network during glioma differentiation therapy. We showed that the increase in noise due to the ultrasensitive response of cyclin D1 in response to drug treatment can induce bifurcation and heterogeneous responses in glioma differentiation. As such, this noise may reduce drug efficacy in the induction of glioma differentiation. Our model further demonstrated that a feedback loop of cyclin D1 activation can increase the variability in signal transduction and phenotypic transition. The results suggest that interventions inhibiting cyclin D1 feedback could help to enhance drug-induced differentiation efficiency in a noisy environment during glioma differentiation therapy.

## Results

### Ultrasensitive response of cyclin D1 controls drug-induced glioma differentiation

**Fig. 1:**
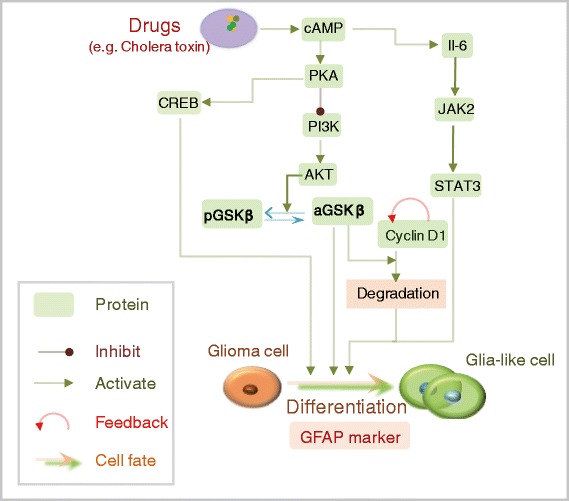
Signaling network of drug-induced glioma differentiation. Signaling pathways involved in the regulation of glioma differentiation during glioma differentiation. Elevation of cAMP level by cholera toxin (CT), can induce glioma cell differentiation, which is mediated by CREB phosphorylation at Ser-133 in a PKA dependent manner [2]. cAMP/PKA signaling can also inhibit the PI3K/AKT pathway, leading to activation of the downstream molecule GSK-3β and subsequent degradation of cyclin D1 [3]. Additionally, the IL-6/JAK2/STAT3 pathway, which is activated by increased cAMP levels, is also involved in glioma cell differentiation [4]. Glial fibrillary acidic protein (GFAP) is used as a reliable marker for evaluating the differentiation of glioma cells

**Fig. 2:**
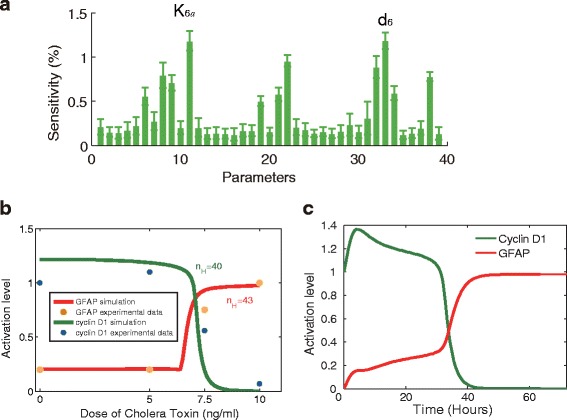
Ultrasensitive response of cyclin D1 controls the phenotypic transition of drug-induced glioma differentiation. **a** The sensitivity analysis revealed that cyclin D1 critically regulates glioma differentiation. **b** Activation levels of cyclin D1 and GFAP under treatment with increasing doses of CT for 48 h. Lines represent simulations (*green* - cyclin D1; *red* - GFAP), and dots are experimental data (*blue* - cyclin D1; *brown* - GFAP). The apparent Hill coefficients (*n*_*H*_) of the simulated dose-response curves of cyclin D1 and GFAP are 40 and 43, respectively, indicating an ultrasensitive response of glioma differentiation to drug treatment. **c** Time-courses of cyclin D1 (*green*) and GFAP (*red*) following drug treatment (CT = 10 ng/ml)

Based on a validated set of parameter values obtained by fitting experimental data [5], we performed parameter a sensitivity analysis (see Methods) to investigate which of the parameters in the developed signaling network model (Fig. 1) were most sensitive or critical for glioma differentiation. The value of each parameter was increased by 5 % from its estimated value, and the time-averaged percent change in the level of GFAP was then obtained. The computations were repeated 20 times, and the mean value and standard deviation were then calculated (Fig. 2a). It was observed that among all of the parameters, two cyclin D1-associated parameters, *K*_6*a*_ (the Michaelis constant for self-feedback of cyclin D1), and *d*_6_ (the deactivation rate of cyclin D1 induced by active GSK3β) were the most sensitive to small variations. These sensitive parameters indicate the critical role of cyclin D1 in regulating glioma differentiation.

The quantified experimental data [2, 3] showed the dose responses of cyclin D1 and GFAP to CT. Our simulation (Fig. 2b) using the validated model further indicated a rapid decrease in the response of cyclin D1 as well as a steep rise in the response of GFAP to increasing CT stimulation within a narrow range (6 to 7 ng/ml). This is a characteristic indication of “ultrasensitivity” in the dose-response relationship [10, 11]. Therefore, we employed an “apparent Hill coefficient” [12, 13] to quantitatively evaluate whether the response of glioma differentiation is ultrasensitive to CT. This Hill coefficient is defined by the following equation [12, 13]:

1 $$ H= \ln 81/ \ln \left(E{C}_{90}/E{C}_{10}\right), $$

where *EC*_90_ and *EC*_10_ represent the stimuli that generate 90 and 10 % of the maximal response, respectively. The apparent Hill coefficients of the simulated dose-response curves for cyclin D1 and GFAP with respect to CT were 40 and 43, respectively (Fig. 2b), indicating strong ultrasensitivity in the response of glioma differentiation to drug treatment. These results demonstrated that the dynamics of differentiation-associated protein activation (i.e. cyclin D1 and GFAP activation) might be regulated by an ultrasensitive mechanism through which low drug levels induce minimal cyclin D1 degradation and GFAP activation but degradation/activation is strongly induced once the drug dose increases above a threshold. Fig. 2c further shows the time-course of cyclin D1 and GFAP following drug treatment (CT = 10 ng/ml) in the deterministic model.

We then experimentally tested the regulatory role of cyclin D1 in the differentiation of human malignant glioma cells (U87-MG cells) by silencing CCND1, which encodes cyclin D1 protein, and pharmacologically downregulating or inhibiting cyclin D1. We selected the most efficient siRNA fragment 003 to knockdown CCND1 (Additional file 1: Figure S1a). Knockdown of CCND1 induced GFAP expression, accompanied by downregulation of proliferating cell nuclear antigen (PCNA, a marker for cell proliferation) (Additional file 1: Figure S1b). Additionally, we used the cAMP analogue 8-CPT-cAMP to mimic the inhibitory effect of cAMP signal activators such as cholera toxin and forskolin on cyclin D1 protein [2, 14]. As shown in Additional file 1: Figure S1c, 8-CPT-cAMP triggers downregulation of cyclin D1, leading to a significant increase in GFAP, but a decrease in PCNA. To further demonstrate the regulatory role of cyclin D1 in the glia-fate induction of glioma cells, we introduced a functional pharmacologic inhibitor of CDK4 and 6 which bind to cyclin D1 to form a complex required for G1-S cell cycle phase progression [15]. The CDK4/6 inhibitor induced the same changes in GFAP and PCNA as siCCND1 and 8-CPT-cAMP (Additional file 1: Figure S1c). Moreover, all of the applied strategies targeting cyclin D1 were able to transform the polygonal bodies of U87-MG cells into a glia-like morphology with dramatically extended processes (Additional file 1: Figure S1d). These data demonstrate the role of cyclin D1 in glioma differentiation, in accordance with the characteristics of our model.

### Noise-induced heterogeneous response of glioma differentiation

**Fig. 3:**
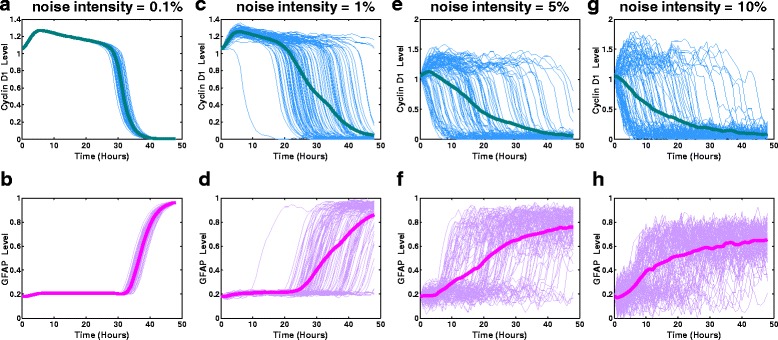
Heterogeneous response of cyclin D1 and GFAP in glioma cells simulated using the ANM model. The simulated cells were subjected to continuous stimulation with CT (10 ng/ml) for 48 h. The stochastic evolution of cyclin D1 and GFAP activity was simulated with different noise intensities (0.1 % (**a**, **b**), 1 % (**c**, **d**), 5 % (**e**, **f**) and 10 % (**g**, **h**), respectively) of signal variation

**Fig. 4:**
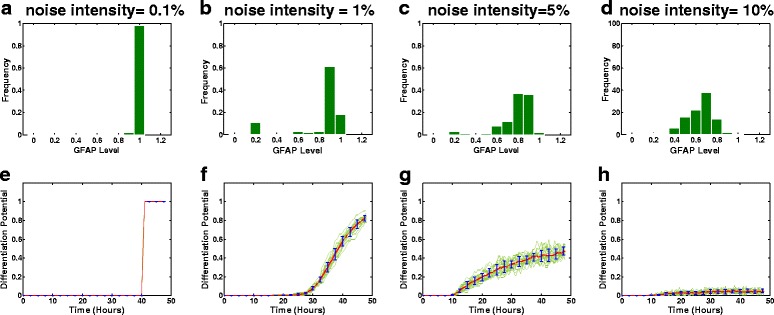
Stochastic cellular responses of glioma cells to differentiation therapy simulated using the ANM model with different noise intensities. The noise intensity was set at 0.1, 1, 5 or 10 %. The upper panel **a**, **b**, **c**, **d** shows the probabilistic distribution of GFAP at 48 h during drug treatment (CT = 10 ng/ml) at different noise intensities. The lower panel **e**, **f**, **g**, **h** shows the stochastic temporal evolution of the differentiation potential of the glioma cell population at different noise intensities

**Fig. 5:**
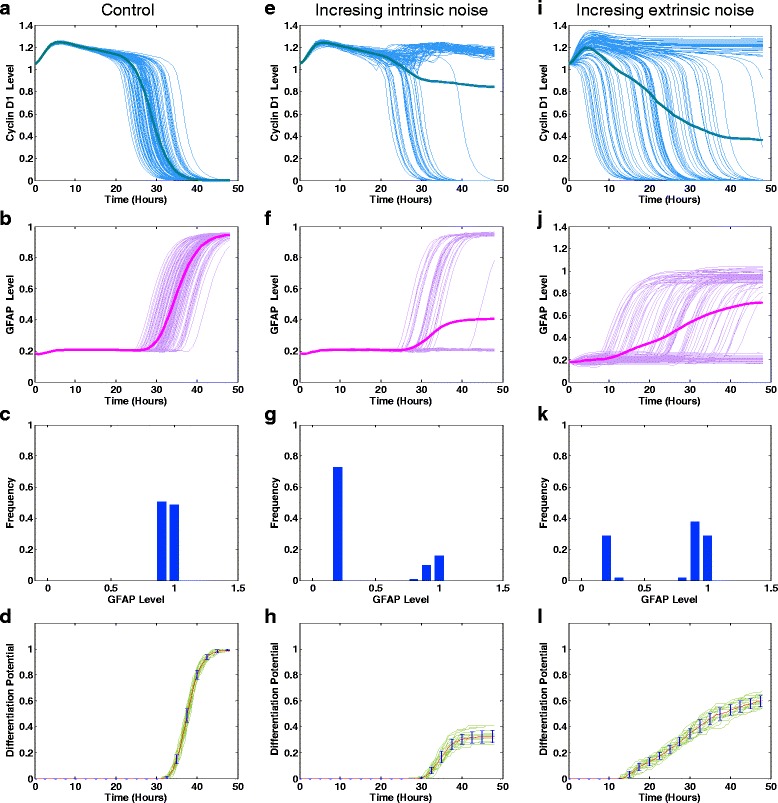
Effects of intrinsic and extrinsic noises on the molecular and cellular responses simulated using the CLE model. **a**-**d** Control group: the intrinsic noise has a standard deviation of $1/\sqrt{V}$ =0.001, and the extrinsic noise has a standard deviation of *λ* = 0.001; **e**-**f** increasing strength of intrinsic noise ($1/\sqrt{V}$ =0.01); **i**-**l** increasing strength of extrinsic noise (*λ* = 0.01). The stochastic temporal responses of cyclin D1 and GFAP, the distribution of GFAP levels and the differentiation potential of glioma cells evaluated at 48 h were simulated

Here, we investigated the stochastic dynamics of cyclin D1 and GFAP concentrations in a noisy environment. The simulations using the ANM model (see Methods) (Fig. 3) showed the temporal evolution of cyclin D1 and GFAP activation at different noise intensities (σ in the ANM model is set to 0.1, 1, 5 or 10 %, as in Ref. [16]). Additional file 1: Figure S2 shows good agreement between the experimental data and simulated GFAP levels at a 5 % noise intensity. We found that increasing the noise intensity impacted the dynamics and distributions of both the cyclin D1 and GFAP responses (Fig. 3, Fig. 4a–d). Meanwhile, the simulations using the CLE model (Fig. 5e–f, i–j) further demonstrated that with an increase of the intrinsic/extrinsic noise intensity, not all trajectories of cyclin D1 are downregulated by CT, and not all trajectories of GFAP are upregulated. This implies that increasing noise strength in signal transduction can induce bifurcation of cyclin D1 degradation, which renders some glioma cells insensitive to drug treatment and induces heterogeneous activation of GFAP. These results indicate that noise can modulate the ultrasensitive response of cyclin D1 and induce heterogeneous drug responses of glioma cells during differentiation therapy.

### Increasing noise leads to a reduction of the differentiation efficiency

We next examined the noise-induced qualitative changes in cyclin D1 and GFAP in glioma cells. As simulated using the ANM (Fig. 4a–d) and CLE (Fig. 5c, g, k) models, an increase in the noise intensity affected the probabilistic distribution of GFAP, indicating that the frequency of the higher levels of GFAP equilibrium decreases with the increase of noise intensity.

To understand how noise impacts the dynamics of the drug-treated glioma cell population, we define the differentiation potential (*D*) as the percent differentiation of glioma cells induced during drug treatment. That is,

2 $$ D(t)={\displaystyle \int u(x){p}_{GFAP}\left(x,t\right)dx}, $$

where *p*_*GFAP*_(*x*, *t*) is the probability distribution function (PDF) describing the concentration (*x*) of GFAP across a population, and *u*(*x*) is a microscopic indictor function describing the effect of the drug on the differentiation of glioma cells at a given GFAP level. Note that *u*(*x*) may be defined as a Heaviside function, such that glioma cells are able to differentiate only if GFAP levels exceed a critical value, *x*_*c*_. That is, *u*(*x*) =1 if *x* > *x*_*c*_ and *u*(*x*) =0 otherwise. *x*_*c*_ is set to 0.8 in this work.

Figure 4e-h shows 20 realizations (green lines) of the stochastic temporal evolution of the differentiation potential of glioma cells simulated using the ANM model. The red line represents the mean value, and the standard deviations are shown with blue error bars at different time points in each situation. As the noise intensity increases, the differentiation potential is significantly reduced, indicating that drug efficacy in inducing glioma differentiation is decreased. These results imply that intra- or extracellular noise or, more generally, complex signaling interference, could reduce the differentiation efficiency of drug-treated glioma cells during differentiation therapy.

**Fig. 6:**
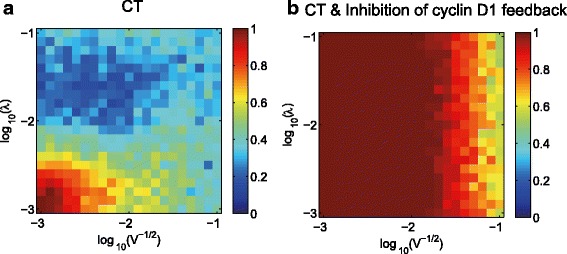
Differentiation potential simulated using the CLE model with a wide range of intrinsic and extrinsic noise strengths. The combined intrinsic and extrinsic noise strength in the range of 10^-3^ to 10^-1^ was examined. **a** The differentiation potential of CT-treated glioma cells. **b** The differentiation potential of glioma cells treated with CT combined with inhibition of cyclin D1 feedback. The results were evaluated at 48 h. The inhibition of cyclin D1 feedback was simulated by increasing the value of the Michaelis constant (*K*_*6a*_) of the cyclin D1 feedback loop by 10-fold in the CLE model

We also used the CLE model to investigate the effects of intrinsic and extrinsic noise on the differentiation potential. Figure 5 shows the stochastic temporal responses of cyclin D1 and GFAP, the distribution of GFAP levels and the differentiation potential of glioma cells evaluated after 48 h of drug treatment (CT = 10 ng/ml). In the control group (Fig. 5a–d), the intrinsic noise has a standard deviation of $1/\sqrt{V}$ =0.001, and the extrinsic noise has a standard deviation of *λ* = 0.001. We then increased the strength of intrinsic noise ($1/\sqrt{V}$ =0.01) (Fig. 5e–h). When these two groups were compared, we found that elevation of the strength of intrinsic noise resulted in the increased heterogeneity of molecular and cellular responses and a decreased differentiation potential. A similar effect was observed for extrinsic noise, as shown in Fig. 5i–l, where the strength of extrinsic noise was increased from *λ* = 0.001 to *λ* = 0.01, which also resulted in a decrease in the differentiation potential. Furthermore, a comprehensive investigation of the effects of the combined strength of intrinsic and extrinsic noise over a wide range (Fig. 6a) clearly showed that increasing the intrinsic and/or extrinsic noise leads to a reduction of the differentiation efficiency.

### Inhibition of cyclin D1 feedback leads to enhancement of the differentiation efficiency

Positive feedback of cyclin D1 activation (e.g., through cyclin D1 auto-activation or the cyclin D1/CDK4-6/Rb/E2F/cyclin D1 feedback loop [17, 18]) has been demonstrated to be involved in glioma differentiation [5]. We investigated whether inhibiting the cyclin D1 feedback loop could enhance the differentiation efficiency. The effect of interventions blocking cyclin D1 feedback is simulated by increasing the value of the Michaelis constant (*K*_*6a*_) in feedback loop by 2-, 5- or 10-fold. We first used both the ANM model (Additional file 1: Figure S4) and the CLE model (Additional file 1: Figure S5) to simulate the stochastic responses of cyclin D1 and GFAP in CT-treated cells, with strong or weak cyclin D1 feedback. The noise intensity in the ANM model was set to 5 % to illustrate a typical simulation (Additional file 1: Figure S2). The strength of intrinsic noise was $1/\sqrt{V}$ =0.01, and the strength of extrinsic noise was *λ* = 0.01. Both Additional file 1: Figure S4 and Additional file 1: Figure S5 show that, compared with the single CT treatment, the combining therapy using CT and inhibition of cyclin D1 feedback results in a rapid degradation of cyclin D1 and the consistent increase of GFAP activity, with decreased heterogeneities in both cyclin D1 and GFAP responses. We used other methods to investigate the role of the cyclin D1 feedback loop as well: (1) decreasing the Hill coefficient of feedback (*n*_*2*_) by 0.2- or 0.5- fold and (2) decreasing the activation rate of the self-feedback of cyclin D1 by 0.2- and 0.5- fold in the model. The results obtained using both of these two methods were consistent with the previous findings.

We also ran the CLE model with a large range of intrinsic and extrinsic noise strengths (from 10^-3^ to 10^-1^) to examine the effect of inhibition of cyclin D1 feedback on the differentiation potential (Fig. 6). The differentiation potential of CT-treated cells in the presence of strong (Fig. 6a) and weak (Fig. 6b) cyclin D1 feedback were examined. Comparison of these two situations demonstrated that inhibition of cyclin D1 feedback enhances the differentiation potential of CT-treated glioma cells. These results imply that inhibiting the cyclin D1 feedback loop might help to reduce noise-induced drug resistance and improve the anti-cancer effects of glioma differentiation therapy.

## Discussion

In this study, we developed a stochastic model of the signaling pathways involved in glioma differentiation therapy to analyze the functional role of noise in the drug response of glioma cells to differentiation inducers. Our analysis indicated that noise can interfere with the ultrasensitive response of cyclin D1 and reduce the differentiation efficiency by inducing heterogeneous responses of glioma cells to drugs. The ultrasensitive response of cyclin D1 is brought about through positive feedback, as inhibiting the feedback loop of cyclin D1 results in rapid degradation of cyclin D1, even without CT treatment. As such, the ultrasensitive mechanism involved in the cyclin D1 response to CT would not exist if this positive feedback loop were blocked. In addition, our simulation suggested that the combination of differentiation therapies with cyclin D1 feedback inhibition might improve therapeutic efficacy.

Noise is an inherent feature of dynamic and stochastic biological systems (e.g., cancer). Whether noise is “beneficial” or “harmful” to a cellular function is an interesting topic, about which there has been some controversy in previous studies [19]. Functional noise is thought to be based on mechanisms intrinsic to network structure or biological systems themselves. In this study, we revealed that cyclin D1 ultrasensitivity might result in qualitative modification of the probability distribution of glioma cell differentiation due to the stochastic noise in molecular processes [20]. This noise may include statistical mechanical fluctuations in protein activation (intrinsic noise) [21] and extracellular micro-environmental perturbations (extrinsic noise) [20]. In this context, innate intra- or extracellular noise might be utilized by glioma tumor cells to resist drugs, which may reflect the inherent adaptation characteristics and acquired fitness of cancers.

Drug resistance is often a major cause of the failure of chemotherapy [22]. The paradigms surrounding drug-resistance mechanisms have focused on understanding drug resistance at the molecular, cellular, and micro-environmental levels. A well established paradigm for the mechanisms underlying drug resistance is that a variety of newly acquired genetic and epigenetic modifications can render tumor cells insensitive to therapeutic agents [23]. Another paradigm that is more often observed in various cancer studies involving targeted therapy is that subtle posttranslational activations of signaling pathways that bypass the stress of the therapeutic target can modulate the expression patterns of oncogenes [24–26]. It has been demonstrated that micro-environmental adaptations [27] play an important role in promoting the rapid emergence of acquired drug resistance due to the drug-induced secretion of various resistance factors from tumor cells [28, 29]. These studies have provided us with abundant information allowing us to understand and potentially overcome drug resistance.

Our modeling experiments highlighted the possibility that the dynamic and stochastic features of post-translational modifications [30] of protein activation might also reduce drug efficacy, thus facilitating drug resistance, independent of genetic mutations. The post-translational mechanism underlying the activity of the cyclin D1 protein revealed in this study is consistent with experimental data (referring to Fig. 5 in Ref. [3], showing that cellular cyclin D1 protein levels are remarkably reduced, while the mRNA levels of cyclin D1 remain unaltered following treatment with CT).

The ANM model includes constant noise that is independent of protein concentrations. The noise term in the ANM model does not take into account the origin of the randomness in biochemical reactions and does not have the capacity to describe intrinsic fluctuations, which is not sufficient in many cases as discussed in Refs. [31, 32]. Additionally, as a multiplicative from of noise, the noise term in the CLE model that approximates the chemical master equation depends on protein concentrations, and therefore appropriately describes the intrinsic noise coming from biochemical reactions. The difference between the ANM and CLE models might lead to discrepancies in their simulation results. For example, in the present study, when the inhibition of cyclin D1 feedback was simulated, the CLE model clearly showed a significantly higher steady-state GFAP level (Additional file 1: Figure S5b) compared with the wild-type (Additional file 1: Figure S5a), while in ANM model, additive noise introduced fluctuations as only small oscillations of the steady-state of cyclin D1 and GFAP, which triggered transition of the steady-states in some trajectories of cyclin D1 from high to low and thus, those of GFAP from low to high (Additional file 1: Figure S4a), due to the irreversible “one-way switch” mechanism [5]. Therefore, in the ANM model, the averaged steady-state of GFAP in the wild-type (Additional file 1: Figure S4a) was almost as high as that observed when cyclin D1 feedback was inhibited (Additional file 1: Figure S4b).

To further verify the correlation between the level of noise in the cyclin D1 protein concentration and the differentiation rate of glioma cells, we will utilize time-lapse microscopy and a customized cell tracking system to monitor the degradation of cyclin D1 and the levels of the differentiation marker GFAP in thousands of individual cells under exposure to cholera toxin at a series of effective doses (5 to 10 ng/ml) [2]. Specifically, the detailed design of the experimental procedure will be as follows: (1) Cell lines. C6 rat glioma cells will be obtained from the American Type Culture Collection (Manassas, VA, USA) and maintained in DMEM (Invitrogen, Grand Island, NY, USA) supplemented with 10 % FBS in a humidified atmosphere of 5 % CO_2_ at 37 °C [2]. (2) Constructs. CCND1 cDNA-red fluorescent protein (RFP) fusions and GFAP cDNA-green fluorescent protein (GFP) fusions will be constructed and transduced into C6 cells. After sorting via flow cytometry, pure populations expressing the desired fluorescent reporters will be obtained and used to establish stable cell strains expressing cyclin D1 and GFAP with fluorescent proteins. (3) Drug treatment. Cells from the stable cell strains will be exposed to cholera toxin (Sigma, St Louis, MO, USA) at effective concentrations of 5, 6, 7, 8, 9 and 10 ng/ml and then subjected to continuous image capture for 48 h [2]. (4) Time-lapse microscopy and image processing. Images will be obtained using an IXMicro microscope (Molecular Devices, Sunnyvale, CA, USA). Each image showing a red fluorescent signal from cyclin D1 and a green fluorescent signal from GFAP will be processed using a low-pass Gaussian filter and the Matlab function *regionprops* followed by procedures similar to those described in Ref. [33]. The algorithm will be performed in MATLAB (MathWorks).

Through the above procedures, the temporal changes in cyclin D1 and GFAP will be measured. We will then calculate the coefficient of variation (CV) for cyclin D1 in response to different doses of cholera toxin. Additionally, we will calculate the correlation coefficient between the CV of cyclin D1 and GFAP level after 24 h or 48 h under the corresponding conditions. If this correlation coefficient is close to -1, then the experimental data are consistent with the model prediction that increasing noise reduces glioma differentiation efficiency.

In our ongoing work, we will further investigate more detailed molecular regulatory networks [34, 35] underlying the feedback loop of cyclin D1 activation. First, the identification of such molecules [36] will advance our understanding of the molecular mechanisms underlying resistance to differentiation therapy. Second, when combined with differentiation therapy, the identified proteins (we will specify them) may be candidate targets for reducing drug resistance [37].

## Conclusions

We have investigated the functional role of stochastic noise in the drug response and differentiation efficiency during cancer differentiation therapy based on an experimentally validated model. Our stochastic modeling of glioma differentiation therapy as a realistic case study demonstrated that increased noise can modulate the ultrasensitivity of cyclin D1 activity and decrease the efficiency of drug-induced glioma differentiation. Moreover, the combination of differentiation-inducible drugs and inhibition of cyclin D1 feedback can enhance the differentiation efficiency of glioma cells. These results advance our understanding of the relationship between noise and drug efficacy in glioma differentiation. Additionally, our study indicates the potential benefit of targeting the dynamics of some critical molecules during cancer therapy to increase anti-cancer effects.

## Methods

### Cell lines and agents

Human glioblastoma U87-MG cells were obtained from the American Type Culture Collection (Manassas, VA). 8-(4-Chlorophenylthio)-adenosine-3′5′-cyclic monophosphate sodium salt (8-CPT-cAMP) was purchased from the BioLogLife Science Institute (Bermen, Germany) and the CDK4/6 inhibitor PD 0332991 was purchased from Selleckchem (Houston, TX).

### Gene silencing using CCND1 siRNA

The siRNA fragments 001, 002 and 003 targeting human CCND1 were purchased from Sigma-Aldrich (St Louis, MO), and the sequences of these fragments were described as follows: siRNA 001-CCACAGAUGUGAAGUUCAUdTdT and AUGAACUUCACAUCUGUGGdTdT; siRNA 002-GCAUGUUCGUGGCCUCUAAdTdT and UUAGAGGCCACGAACAUGCdTdT; siRNA 003-GUAAGAAUAGGCAUUAACAdTdT and UGUUAAUGCCUAUUCUUACdTdT. CCND1 siRNA was transfected into U87-MG cells using the Lipofectamine™ RNAiMAX reagent (Invitrogen, Carlsbad, California). After 1, 2 and 3 days, proteins from the transfected cells were subjected to western blot analysis and the protein levels of cyclin D1, GFAP and PCNA were evaluated with specific antibodies.

### Western blot analysis

U87-MG cells were treated with CCND1 siRNA, 8-CPT-cAMP or CDK4/6 inhibitor for different times. Total proteins were extracted with the Mammalian Protein Extraction Reagent (Pierce, Rockford, IL, USA) and then subjected to measurement of the protein concentration with the BCA Protein AssayKit (Pierce, Rockford, IL, USA). Next, equal amounts of the protein samples were separated via sodiumdodecylsulphate-polyacrylamide gel electrophoresis (SDS-PAGE) and then electrotransferred to a PVDF membrane. Primary antibodies against cyclin D1, GPAP, PCNA (Cell Signaling Technology, Beverly, MA, USA) and Tubulin (Sigma-Aldrich, St Louis, MO) and a horseradish peroxidase-labeled secondary antibody (Cell Signaling Technology, Beverly, MA, USA) were used to recognize these specific proteins. Finally, the proteins were visualized with enhanced chemiluminescence detection reagents (Pierce, Rockford, IL, USA) in an immunoblotting imaging and analysis system (BioRad, CA, USA).

### Additive noise model

As a simple and the easiest approach for incorporating molecular fluctuations in the model [16, 38], an additive noise model (ANM) [39–42] that incorporates an additive noise term into the stochastic differential equation was adopted in this study to simulate stochastic signal transduction in the regulation of glioma differentiation. The ANM model is described by the following equations:

3 $$ dY=F\left(t,Y\right)\cdot dt+\sigma \cdot dW $$

where *Y* = {*y*_*k*_, *k* = 1, ⋯, 10} is a set of random variables describing the activation levels of the molecular components in the signaling pathway. The drift term, *F*(*Y*), in the above model is a matrix consisting of functions that describe chemical reaction rates between molecular components. The symbol σ represents the noise intensity determining the amplitude of noise in the system. The symbol *W* = {*ω*_*k*_(*t*), *k* = 1, ⋯, 10} represents a set of independent Wiener processes or standard Brownian motion, characterized by the following equation:

4 $$ \Delta {\omega}_k={\omega}_k\left(t+\Delta t\right)-{\omega}_k(t)\sim N\left(0,\Delta t\right)=\sqrt{\Delta t}N\left(0,1\right),\;k=1,\cdots, 10. $$

where *N*(0,1) is the unit normal distribution.

The drug-induced activation of the PKA-PI3K-AKT-GSK3β pathway (Fig. 1) is modeled by the following equations (5–9) using Michaelis-Menten kinetics [43] and Hill functions [44]:

5 $$ d\left[PKA\right]={a}_1dt+\frac{V_1\cdot C{T}^{n_1}}{K_1+C{T}^{n_1}}dt-{d}_1\left[PKA\right]dt+{\sigma}_1d{W}_1 $$

6 $$ d\left[ CREB\right]=\frac{V_2\cdot \left[PKA\right]}{K_2+\left[PKA\right]}dt-{d}_2\left[ CREB\right]dt+{\sigma}_2d{W}_2 $$

7 $$ d\left[ PI3K\right]=\frac{1}{1+\left[PKA\right]/{K}_3}dt-{d}_3\left[ PI3K\right]dt+{\sigma}_3d{W}_3 $$

8 $$ d\left[AKT\right]=\frac{V_4\cdot \left[ PI3K\right]}{K_4+\left[ PI3K\right]}dt-{d}_4\left[AKT\right]dt+{\sigma}_4d{W}_4 $$

9 $$ d\left[ pGSK3\beta \right]=\left(\frac{V_5\cdot \left(\left[GSK3{\beta}_T\right]-\left[ pGSK3\beta \right]\right)}{K_5+\left[GSK3{\beta}_T\right]-\left[ pGSK3\beta \right]}\cdot \left[AKT\right]\right)dt-{d}_5\left[ pGSK3\beta \right]dt+{\sigma}_5d{W}_5. $$

The cAMP-PKA mediated activation of the IL-6-JAK2-STAT3 pathway induced by CT is modeled as follows:

10 $$ d\left[IL6\right]=\frac{V_7\cdot \left[PKA\right]}{K_7+\left[PKA\right]}dt-{d}_7\left[IL6\right]dt+{\sigma}_7\cdot d{W}_7 $$

11 $$ d\left[JAK2\right]=\frac{V_8\cdot \left[IL6\right]}{K_8+\left[IL6\right]}dt-{d}_8\left[JAK2\right]dt+{\sigma}_8\cdot d{W}_8 $$

12 $$ d\left[ STAT3\right]=\frac{V_9\cdot \left[JAK2\right]}{K_9+\left[JAK2\right]}dt-{d}_9\left[ STAT3\right]dt+{\sigma}_9\cdot d{W}_9. $$

The stochastic kinetics of cyclin D1, balanced by its activation and degradation, is modeled as follows:

13 $$ d\left[ CyclinD1\right]=\left({V}_6\cdot \frac{{\left[ CyclinD1\right]}^{n_2}}{K_{6a}^{n_2}+{\left[ CyclinD1\right]}^{n_2}}\right)dt-\left({d}_6\cdot \frac{\left[ aGSK3\beta \right]}{K_{6b}+\left[ aGSK3\beta \right]}\cdot \left[ CyclinD1\right]\right)dt+{\sigma}_6\cdot d{W}_6 $$

where the first term on the right-hand side describes the activation of cyclin D1 promoted by self-amplification or a positive feedback loop for cyclin D1 [17, 18] that has been validated in our previous study [5]. *V*_6_ is the maximal activation rate of cyclin D1, and *K*_6*a*_ is the Michaelis constant. *n*_2_ is the Hill coefficient. The second term on the right-hand side of the above equation describes the deactivation of cyclin D1 induced by active GSK3β, which can trigger cyclin D1 translocation and degradation. *d*_6_ is the dephosphorylation rate of cyclin D1, and *K*_6*b*_ is the Michaelis constant for GSK3β-induced cyclin D1 degradation. *W*_6_ is standard Brownian motion, and *σ*_6_ is a diffusion coefficient.

As a reliable marker of the differentiation of glioma cells, GFAP is regulated by CREB, STAT3 and active GSK3β [2, 4]. Degradation of cyclin D1 is required for the differentiation of glioma cells [3]. Therefore, the stochastic dynamics of GFAP can be modeled using the following equation:

14 $$ d\left[ GFAP\right]={f}_0\left(\left[ CyclinD1\right]\right)\cdot \left(\frac{V_{10 ab}\cdot \left[ CREB\right]}{K_{10a}+\left[ CREB\right]}\cdot \frac{\left[ STAT3\right]}{K_{10b}+\left[ STAT3\right]}+\frac{V_{10c}\cdot {\left[ aGSK3\beta \right]}^{n3}}{K_{10c}+{\left[ aGSK3\beta \right]}^{n3}}\right)dt-{d}_{10}\left[ GFAP\right]dt+{\sigma}_{10}d{W}_{10} $$

where ${f}_0\left(\left[ CyclinD1\right]\right)={\left(\frac{C-\left[ CyclinD1\right]}{C}\right)}^{+}$ with *C* being the maximal value of the steady-state of cyclin D1, and $$ {(x)}^{+}=\left\{\begin{array}{l}x,x>0\\ {}0,x\le 0\end{array}\right. $$. As the upregulation of cyclin D1 is indispensable in the cell cycle and cell proliferation, it is only when cyclin D1 is downregulated that glioma cells can begin to differentiate and, thus, that GFAP can be unregulated. Therefore cyclin D1 is modeled as being dominant in GFAP regulation. The involvement of CREB, STAT3 and GSK3β in the regulation of GFAP is modeled by determining the best fit of the model structure to the experimental data under various conditions [5]. The last two terms in the above equation describe the degradation and fluctuations of GFAP.

### Chemical-Langenvin-Equation (CLE) model

The variation in signal transduction arises from various sources, including intrinsic and extrinsic factors [45]. The intrinsic factors include statistical mechanical fluctuations in the diffusion and binding of the molecules involved in protein activation. The extrinsic factors include fluctuations in the extracellular environment [46], the stochasticity of gene expression [47], variations in the epigenetic state [48], and different levels of molecular machines [45, 49], etc.

To examine the effects of intrinsic noise on glioma differentiation, we also employed the CLE model to simulate the stochastic molecular responses of glioma cells to drug treatment, and to extend the predictions of the ANM model. Based on the deterministic ODE model, the “white-noise form” Langevin equations [50] are formulated as follows:

15 $$ \frac{d\left[PKA\right]}{dt}=\left({a}_1+\frac{V_1\cdot C{T}^{n_1}}{K_1+C{T}^{n_1}}\right)-{d}_1\left[PKA\right]+\frac{1}{\sqrt{V}}\left[\sqrt{a_1+\frac{V_1\cdot C{T}^{n_1}}{K_1+C{T}^{n_1}}}{\zeta}_1(t)-\sqrt[]{d_1\left[PKA\right]}{\zeta}_2(t)\right] $$

16 $$ \frac{d\left[ CREB\right]}{dt}=\frac{V_2\cdot \left[PKA\right]}{K_2+\left[PKA\right]}-{d}_2\left[ CREB\right]+\frac{1}{\sqrt{V}}\left[\sqrt[]{\frac{V_2\cdot \left[PKA\right]}{K_2+\left[PKA\right]}}{\zeta}_3(t)-\sqrt[]{d_2\left[ CREB\right]}{\zeta}_4(t)\right] $$

17 $$ \frac{d\left[ PI3K\right]}{dt}=\frac{1}{1+\left[PKA\right]/{K}_3}-{d}_3\left[ PI3K\right]+\frac{1}{\sqrt{V}}\left[\sqrt[]{\frac{1}{1+\left[PKA\right]/{K}_3}}{\zeta}_5(t)-\sqrt[]{d_3\left[ PI3K\right]}{\zeta}_6(t)\right] $$

18 $$ \frac{d\left[AKT\right]}{dt}=\frac{V_4\cdot \left[ PI3K\right]}{K_4+\left[ PI3K\right]}-{d}_4\left[AKT\right]+\frac{1}{\sqrt{V}}\left[\sqrt[]{\frac{V_4\cdot \left[ PI3K\right]}{K_4+\left[ PI3K\right]}}{\zeta}_7(t)-\sqrt[]{d_4\left[AKT\right]}{\zeta}_8(t)\right] $$

19 $$ \begin{array}{l}\frac{d\left[ pGSK3\beta \right]}{dt}=\left(\frac{V_5\cdot \left(\left[GSK3{\beta}_T\right]-\left[ pGSK3\beta \right]\right)}{K_5+\left[GSK3{\beta}_T\right]-\left[ pGSK3\beta \right]}\cdot \left[AKT\right]\right)-{d}_5\left[ pGSK3\beta \right]\\ {}\kern5.75em +\frac{1}{\sqrt{V}}\left[\sqrt[]{\left(\frac{V_5\cdot \left(\left[GSK3{\beta}_T\right]-\left[ pGSK3\beta \right]\right)}{K_5+\left[GSK3{\beta}_T\right]-\left[ pGSK3\beta \right]}\cdot \left[AKT\right]\right)}{\zeta}_9(t)-\sqrt[]{d_5\left[ pGSK3\beta \right]}{\zeta}_{10}(t)\right]\end{array} $$

20 $$ \frac{d\left[IL6\right]}{dt}=\frac{V_7\cdot \left[PKA\right]}{K_7+\left[PKA\right]}-{d}_7\left[IL6\right]+\frac{1}{\sqrt{V}}\left[\sqrt[]{\frac{V_7\cdot \left[PKA\right]}{K_7+\left[PKA\right]}}{\zeta}_{11}(t)-\sqrt[]{d_7\left[IL6\right]}{\zeta}_{12}(t)\right] $$

21 $$ \frac{d\left[JAK2\right]}{dt}=\frac{V_8\cdot \left[IL6\right]}{K_8+\left[IL6\right]}-{d}_8\left[JAK2\right]+\frac{1}{\sqrt{V}}\left[\sqrt[]{\frac{V_8\cdot \left[IL6\right]}{K_8+\left[IL6\right]}}{\zeta}_{13}(t)-\sqrt[]{d_8\left[JAK2\right]}{\zeta}_{14}(t)\right] $$

22 $$ \frac{d\left[ STAT3\right]}{dt}=\frac{V_9\cdot \left[JAK2\right]}{K_9+\left[JAK2\right]}-{d}_9\left[ STAT3\right]+\frac{1}{\sqrt{V}}\left[\sqrt{\frac{V_9\cdot \left[JAK2\right]}{K_9+\left[JAK2\right]}}{\zeta}_{15}(t)-\sqrt[]{d_9\left[ STAT3\right]}{\zeta}_{16}(t)\right] $$

23 $$ \begin{array}{l}\frac{d\left[ CyclinD1\right]}{dt}=\left({V}_6\cdot \frac{{\left[ CyclinD1\right]}^{n_2}}{K_{6a}^{n_2}+{\left[ CyclinD1\right]}^{n_2}}\right)-\left({d}_6\cdot \frac{\left[ aGSK3\beta \right]}{K_{6b}+\left[ aGSK3\beta \right]}\cdot \left[ CyclinD1\right]\right)\\ {}\kern5.5em +\frac{1}{\sqrt{V}}\left[\sqrt[]{\left({V}_6\cdot \frac{{\left[ CyclinD1\right]}^{n_2}}{K_{6a}^{n_2}+{\left[ CyclinD1\right]}^{n_2}}\right)}{\zeta}_{17}(t)-\sqrt[]{\left({d}_6\cdot \frac{\left[ aGSK3\beta \right]}{K_{6b}+\left[ aGSK3\beta \right]}\cdot \left[ CyclinD1\right]\right)}{\zeta}_{18}(t)\right]\end{array} $$

24 $$ \begin{array}{l}\frac{d\left[ GFAP\right]}{dt}={f}_0\left(\left[ CyclinD1\right]\right)\cdot \left(\frac{V_{10 ab}\cdot \left[ CREB\right]}{K_{10a}+\left[ CREB\right]}\cdot \frac{\left[ STAT3\right]}{K_{10b}+\left[ STAT3\right]}+\frac{V_{10c}\cdot {\left[ aGSK3\beta \right]}^{n3}}{K_{10c}+{\left[ aGSK3\beta \right]}^{n3}}\right)-{d}_{10}\left[ GFAP\right]\\ {}\kern4.5em +\frac{1}{\sqrt{V}}\left[\sqrt[]{f_0\left(\left[ CyclinD1\right]\right)\cdot \left(\frac{V_{10 ab}\cdot \left[ CREB\right]}{K_{10a}+\left[ CREB\right]}\cdot \frac{\left[ STAT3\right]}{K_{10b}+\left[ STAT3\right]}+\frac{V_{10c}\cdot {\left[ aGSK3\beta \right]}^{n3}}{K_{10c}+{\left[ aGSK3\beta \right]}^{n3}}\right)}{\zeta}_{19}-{d}_{10}\left[ GFAP\right]{\zeta}_{20}\right]\end{array} $$

where *V* denotes the total number of molecules of each protein in the above signaling pathway and *ζ*_*i*_ (*i* = 1, ⋯, 20) represents temporally uncorrelated, statistically independent Gaussian white noise, i.e., for each *i*, *j* = 1, ⋯, 20,

25 $$ \left\langle {\zeta}_i(t){\zeta}_j(s)\right\rangle =\left\{\begin{array}{l}0,\ for\ i\ne j\ \\ {}\delta \left(t-s\right),\ for\ i=j\end{array}\right.\ . $$

Furthermore, when extrinsic noise was taken into account, each parameter, *P*_*j*_, in the model was varied as *P*_*j*_(1 + *λε*_*i*_), where *ε*_*i*_ (*i* = 1, ⋯, 39) represents statistically independent Gaussian white noise. *λ* is the strength of the extrinsic noise.

The uniqueness of the solution to the above stochastic differential equations (SDEs) can be guaranteed because their coefficients satisfy certain appropriate growth conditions and local Lipschitz continuity [51]. The biological meaning of the applied parameters and their values are listed in Additional file 1: Table S1. The initial values of the mathematical model are listed in Additional file 1: Table S2. We numerically solved the above SDEs using the Euler-Maruyama method [52]. The simulation was performed in MATLAB R2007b (Math Works, USA). The trajectories of all signaling components in relation to the noise simulated with the CLE model are presented in Additional file 1: Figure S6.

### Parameter sensitivity analysis

Parameter sensitivity analysis is often used to quantitatively explore which parameters are more sensitive in affecting signaling dynamics. The time-dependent sensitivity coefficient of GFAP (model output) at time point *t* with respect to parameter *P*_*j*_ was calculated as follows:

26 $$ {S}_{ij}(t)=\frac{\partial \left[ GFAP\right]}{\partial {P}_j}/\frac{\left[ GFAP\right]}{P_j}\approx \frac{\Delta \left[ GFAP\right]}{\left[ GFAP\right]}/\frac{\Delta {P}_j}{P_j}\;\mathrm{f}\mathrm{o}\mathrm{r}\ \mathrm{small}\;\Delta {P}_j. $$

Time-averaged sensitivities [53] were calculated as shown below to evaluate parameter sensitivity during the entire time course

27 $$ {S}_{ij}={\displaystyle {\int}_0^T\left|{S}_{ij}(t)\right|}dt/T\approx {\displaystyle \sum_{l=1}^L\left|{S}_{ij}\left({t}_l\right)\right|}/L, $$

where {*t*_*l*_, *l* = 1, ⋯ *L*} is an equal partition of [0, *T*], with *L* =100 and *T* = 48 h in the simulation. A small perturbation (*ΔP*_*j*_ =5 %) is imposed for calculating the time-averaged sensitivities of GFAP with respect to the examined parameters.

## Abbreviations

AKT, protein kinase B; cAMP, cyclic adenosine monophosphate; CREB, cAMP-response element binding protein; CT, cholera toxin; GFAP, glial fibrillary acidic protein; GSK-3β, glycogen synthase kinase 3 beta; IL6, Interleukins 6; JAK2, Janus kinase 2; PI3K, phosphoinositide 3-kinase; PKA, protein kinase A; STAT3, signal transducer and activator of transcription 3

## Additional file

Additional file 1: Text S1. Cyclin D1 feedback increases variability in signal transduction and phenotypic transition. Figure S1 Both the downregulation of cyclin D1 expression and inhibition of the function of the cyclin D1/CDK4/6 complex induce the differentiation of human glioblastoma U87-MG cells into glia-like cells. Figure S2 Comparison between the simulated time-course of GFAP levels at a 5 % noise intensity and the experimental data. Figure S3 The coefficient of variation of cyclin D1 and GFAP levels affected by cyclin D1 feedback during glioma differentiation. Figure S4 The effect of inhibition of cyclin D1 feedback simulated with the ANM model. Figure S5 The effect of inhibition of cyclin D1 feedback simulated with the CLE model. Figure S6 Trajectories of all signaling components in relation to noise simulated with the CLE model. Table S1 Parameter values involved in the model. Table S2 Initial values of the mathematical model. (DOC 1244 kb)
