# Supplementary material for: Stochastic modeling suggests that noise reduces differentiation efficiency by inducing a heterogeneous drug response in glioma differentiation therapy
Source: BMC Syst Biol. 2016 Aug 11;10:73. doi: 10.1186/s12918-016-0316-x (PMC4982223; doi:10.1186/s12918-016-0316-x)
Supplement: Additional file 1: — Text S1. Cyclin D1 feedback increases variability in signal transduction and phenotypic transition. Figure S1 Both the downregulation of cyclin D1 expression and inhibition of the function of the cyclin D1/CDK4/6 complex induce the differentiation of human glioblastoma U87-MG cells into glia-like cells. Figure S2 Comparison between the simulated time-course of GFAP levels at a 5 % noise intensity and the experimental data. Figure S3 The coefficient of variation of cyclin D1 and GFAP levels affected by cyclin D1 feedback during glioma differentiation. Figure S4 The effect of inhibition of cyclin D1 feedback simulated with the ANM model. Figure S5 The effect of inhibition of cyclin D1 feedback simulated with the CLE model. Figure S6 Trajectories of all signaling components in relation to noise simulated with the CLE model. Table S1 Parameter values involved in the model. Table S2 Initial values of the mathematical model. (DOC 1244 kb) [file 12918_2016_316_MOESM1_ESM.doc]

Supplementary Material for

Stochastic Modeling Suggests that Noise Reduces Differentiation Efficiency by Inducing a Heterogeneous Drug Response in Glioma Differentiation Therapy

Xiaoqiang Sun^1,2^*, Jiajun Zhang^2^, Qi Zhao^3,4^, Xing Chen^5,6^, Wenbo Zhu^1^*, Guangmei Yan^1^, Tianshou Zhou^2^*

1 Zhong-shan School of Medicine, Sun Yat-Sen University, Guangzhou 510089, China

2 School of Mathematical and Computational Science, Sun Yat-Sen University, Guangzhou 510000, China

3 School of Mathematics, Liaoning University, Shenyang 110036, China

4 Research Center for Computer Simulating and Information Processing of Bio-macromolecules of Liaoning Province, Shenyang 110036, China

5 Academy of Mathematics and Systems Science, Chinese Academy of Sciences, Beijing, 100190, China

6 National Center for Mathematics and Interdisciplinary Sciences, Chinese Academy of Sciences, Beijing, 100190, China

Corresponding authors:

*Xiaoqiang Sun, Ph.D., Assistant Professor.

Email: xiaoqiangsun88@gmail.com; dongbusun@163.com

*Wenbo Zhu, Ph.D., Associate Professor.

Email: zhuwenbo@mail.sysu.edu.cn

*Tianshou Zhou, Ph.D., Professor.

Email: mcszhtsh@mail.sysu.edu.cn

**Text S1 Cyclin D1 feedback increases variability in signaling transduction and phenotypic transition**

A positive feedback of cyclin D1 activation (e.g., through cyclin D1 auto-activation or the cyclin D1/CDK4-6/Rb/E2F/cyclin D1 feedback loop ) has been demonstrated to be involved in glioma differentiation . Therefore we then examined the role of cyclin D1 self-feedback in noise amplification during glioma differentiation. The variability of protein activation and cell differentiation in a population of glioma cells was quantified by the coefficient of variation (*CV*) , which was indicated by the formula listed below:

| $CV=\left(\left\langle g^{2}\right\rangle-\left\langle g\right\rangle^{2}\right)^{1/2}/\left\langle g\right\rangle$ |  |
| --- | --- |

where$g$represents the activation levels of regulatory proteins or differentiation markers in the signaling network, $\left\langle g\right\rangle$ is the mean level of each protein, and $\left(\left\langle g^{2}\right\rangle-\left\langle g\right\rangle^{2}\right)^{1/2}$ is the standard deviation.

**Fig. S2 a, b** show the *CVs* in the cyclin D1 and GFAP levels over time simulated from the ANM model with cyclin D1 feedback. **Fig. S2 c, d** demonstrate the *CV* in the cyclin D1 and GFAP levels over time in the modified model with decreased strength of cyclin D1 feedback (by increasing the value of Michaelis constant (*K_6a_*) in feedback loop by 10 folds). The noise intensity ($\sigma$ in the SDEs model) was set to 5% for a typical simulation. A comparison of these two situations (**Fig. S2 a, b** versus **Fig. S2 c ,d**) illustrates that the overall *CVs* in both cyclin D1 and GFAP levels in wide-type model are greater than that in the modified model without cyclin D1 feedback. These results demonstrate that cyclin D1 feedback could amplify the noise in glioma differentiation.

**Fig S1**


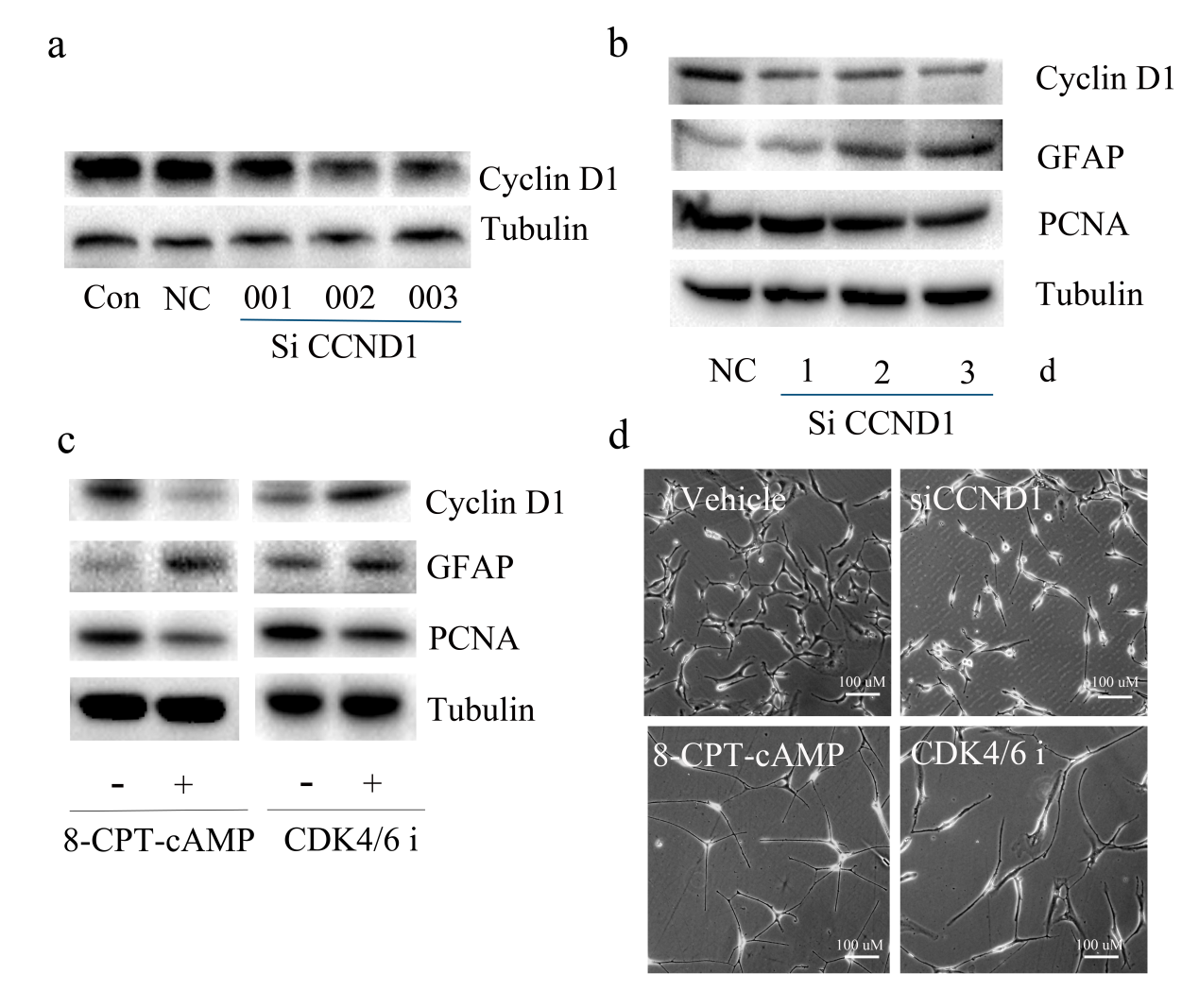


**Figure S1. Both of the downregulation of cyclin D1 expression and inhibition of cyclin D1/CDK4/6 complex function induce the differentiation of human glioblastoma U87-MG cells into glia-like cells.** (a) Efficiency of silencing RNA fragtments (siRNAs) for CCND1. U87-MG cells were incubated with CCND1 siRNAs for 2 days and then were subject to western blot analysis. (b) Immunoblot of cyclin D1, GFAP and PCNA proteins in cells treated with CCND1 siRNA for 1, 2 and 3 days. (c) Immunoblot of cyclin D1, GFAP and PCNA proteins in cells treated with 8-CPT-cAMP or CDK4/6 inhibitor (CDK4/6 i) for 3 days. (c) Morphology of cells treated with CCND1 siRNA, 8-CPT-cAMP or CDK4/6 i for 3 days.

**Fig S2**

**Figure S2. Comparison of time course of GFAP level simulated from ANM model with 5% noise intensity with the experimental data.** Blue line represents the simulation, and res dots are experimental data from .

**Fig S3**

**Figure S3. The coefficient of variations (CVs) in cyclin D1 and GFAP levels affected by cyclin D1 feedback during glioma differentiation**. (**a**, **b**) The CVs in the cyclin D1 level and GFAP level over time with the original strength of cyclin D1 feedback. (**c**, **d**) The CVs in the cyclin D1 level and GFAP level over time with decreased strength of cyclin D1 feedback (by increasing the value of Michaelis constant (*K_6a_*) in feedback loop by 10 folds)

**Fig S4**

**Figure S4**. **The effect of inhibition of cyclin D1 feedback** **simulated with the ANM model.** Stochastic responses of cyclin D1 and GFAP in CT-treated cells, with the original (**a**) or decreased (**b**) strength of cyclin D1 feedback were simulated. Wild-type indicates glioma cells with the original strength of cyclin D1 feedback. The inhibition of cyclin D1 feedback was simulated by increasing the value of Michaelis constant (*K_6a_*) of cyclin D1 feedback loop by 10 folds in the ANM model. The noise intensity was set to 5% to illustrate a typical simulation (See **Fig. S2** for comparison with the experimental data).

**Fig S5**

**Figure S5**. **The effect of inhibition of cyclin D1 feedback simulated with the CLE model.** Stochastic responses of cyclin D1 and GFAP in CT-treated cells, with the original (**a**) or decreased (**b**) strength of cyclin D1 feedback were simulated. Wild-type indicates glioma cells with the original strength of cyclin D1 feedback. The inhibition of cyclin D1 feedback was simulated by increasing the value of Michaelis constant (*K_6a_*) of cyclin D1 feedback loop by 10 folds in the CLE model. The strength of the intrinsic noise is $1/\sqrt{V}$=0.01, and the strength of the extrinsic noise is $\lambda=0.01$.

**Fig S6**

**Figure S6**. **Trajectories of all signaling components to noise simulated with the CLE model**. The simulation shows stochastic responses of PKA, CREB, PI3K, IL6, JAK2, STAT3, GSK3β, cyclinD1 and GFAP in CT-treated cells. The strength of the intrinsic noise is $1/\sqrt{V}$=0.01, and the strength of the extrinsic noise is $\lambda=0.001$.

**Table S1** **The values of parameters in the signaling pathway [3]**.

| **Para No.** | **Symbol** | **Value** | **Description** |
| --- | --- | --- | --- |
| 1 | *V*_1_ | 1.6245 | maximal activation velocity of PKA by Cholera toxin |
| 2 | *K*_1_ | 9.5398 | Michaelis constant of PKA by Cholera toxin |
| 3 | *V*_2_ | 0.6602 | maximal activation velocity of CREB by PKA |
| 4 | *K*_2_ | 0.5351 | Michaelis constant of CREB by PKA |
| 5 | *K*_3_ | 0.7018 | Michaelis constant of PI3K inhibition by PKA |
| 6 | *V*_4_ | 0.1136 | maximal activation velocity of AKT by PI3K |
| 7 | *K*_4_ | 0.4037 | Michaelis constant of AKT by PI3K |
| 8 | *V*_5_ | 0.4449 | maximal phosphorylation velocity of GSK3β by AKT |
| 9 | *K*_5_ | 0.2951 | Michaelis constant of GSK3β by AKT |
| 10 | *V*_6_ | 0.5448 | maximal self-amplification velocity of cyclin D1 |
| 11 | *K*_6a_ | 0.9413 | Michaelis constant of cyclin D1 |
| 12 | *K*_6b_ | 0.8489 | Michaelis constant of cyclin D1 by GSK3β |
| 13 | *V*_7_ | 0.6390 | maximal activation velocity of IL6 by PKA |
| 14 | *K*_7_ | 0.2324 | Michaelis constant of IL6 inhibition by PKA |
| 15 | *V*_8_ | 0.5359 | maximal activation velocity of JAK2 by IL6 |
| 16 | *K*_8_ | 0.2558 | Michaelis constant of JAK2 by IL6 |
| 17 | *V*_9_ | 0.7861 | maximal activation velocity of STAT3 by JAK2 |
| 18 | *K*_9_ | 0.6108 | Michaelis constant of STAT3 by JAK2 |
| 19 | *V*_10ab_ | 0.0841 | maximal activation velocity of GFAP by CREB |
| 20 | *K*_10a_ | 0.1316 | Michaelis constant of GFAP by CREB |
| 21 | *K*_10b_ | 0.1692 | Michaelis constant of GFAP by STAT3 |
| 22 | *V*_10c_ | 0.5249 | maximal activation velocity of GFAP by GSK3β |
| 23 | *K*_10c_ | 0.7371 | Michaelis constant of GFAP by GSK3β |
| 24 | *C* | 1.25 | maximal steady state of cyclin D1 |
| 25 | *V*_11a_ | 0.2975 | maximal activation velocity of PCNA by cyclin D1 |
| 26 | *K*_11a_ | 0.2649 | Michaelis constant of PCNA by cyclin D1 |
| 27 | *V*_11b_ | 0.0834 | maximal activation velocity of PCNA by STAT3 |
| 28 | *K*_11b_ | 0.0172 | Michaelis constant of PCNA by STAT3 |
| 29 | *d*_1_ | 0.4668 | Dephosphorylation rate of PKA |
| 30 | *d*_2_ | 0.5774 | Dephosphorylation rate of CREB |
| 31 | *d*_3_ | 0.4602 | Dephosphorylation rate of PI3K |
| 32 | *d*_4_ | 0.7700 | Dephosphorylation rate of AKT |
| 33 | *d*_5_ | 0.7147 | Dephosphorylation rate of pGSK3β |
| 34 | *d*_6_ | 0.8363 | Dephosphorylation rate of cyclin D1 |
| 35 | *d_7_* | 0.7163 | Dephosphorylation rate of IL6 |
| 36 | *d*_8_ | 0.9704 | Dephosphorylation rate of JAK2 |
| 37 | *d*_9_ | 0.7398 | Dephosphorylation rate of STAT3 |
| 38 | *d*_10_ | 0.4881 | Dephosphorylation rate of GFAP |
| 39 | *d*_11_ | 0.3696 | Dephosphorylation rate of PCNA |
| 40 | *n_1_* | 3 | Hill coefficient of PKA activation induced by cholera toxin |
| 41 | *n_2_* | 10 | Hill coefficient of cyclin D1 feedback |
| 42 | *n_3_* | 8 | Hill coefficient of GFAP activation promoted by active GSK3β |

**Table S2** **Initial values of the mathematical model**.

| **Para No.** | **Symbol** | **Value** | **Description** |
| --- | --- | --- | --- |
| 1 | *y*_1_ | 1.0000 | Initial value of PKA |
| 2 | *y*_2_ | 0.2600 | Initial value of CREB |
| 3 | *y*_3_ | 1.0000 | Initial value of PI3K |
| 4 | *y*_4_ | 1.0000 | Initial value of AKT |
| 5 | *y*_5_ | 0.3000 | Initial value of GSK3β |
| 6 | *y*_6_ | 1.0500 | Initial value of IL6 |
| 7 | *y*_7_ | 0.7239 | Initial value of JAK2 |
| 8 | *y*_8_ | 0.4080 | Initial value of STAT3 |
| 9 | *y*_9_ | 0.6818 | Initial value of cyclin D1 |
| 10 | *y*_10_ | 0.1834 | Initial value of GFAP |

Supplementary Reference

1. Swat M, Kel A, Herzel H: **Bifurcation analysis of the regulatory modules of the mammalian G1/S transition**. *Bioinformatics* 2004, **20**(10):1506-1511.

2. Gérard C, Goldbeter A: **Temporal self-organization of the cyclin/Cdk network driving the mammalian cell cycle**. *Proc Natl Acad Sci U S A* 2009, **106**(51):21643-21648.

3. Xiaoqiang S, Xiaoke Z, Jiajun Z, Tianshou Z, Guangmei Y, Wenbo Z: **Mathematical modeling reveals a critical role for cyclin D1 dynamics in phenotype switching during glioma differentiation**. *FEBS Lett* 2015, **589**(18):2304–2311.

4. Dixit PD: **Quantifying extrinsic noise in gene expression using the maximum entropy framework**. *Biophys J* 2013, **104**(12):2743-2750.
